# Evaluation of Exosomal miRNA as Potential Biomarkers in Cervical Cancer

**DOI:** 10.3390/epigenomes7030016

**Published:** 2023-08-01

**Authors:** Jéssika Aline do Nascimento Medeiros, Ayane Cristine Alves Sarmento, Emanuelly Bernardes-Oliveira, Ronnier de Oliveira, Maysa Eunice Grigorio Bezerra Lima, Ana Katherine Gonçalves, Deyse de Souza Dantas, Janaina Cristiana de Oliveira Crispim

**Affiliations:** 1 Women’s Health Sciences Postgraduate Program, Federal University of Rio Grande do Norte (UFRN), Natal 59012-570, RN, Brazil; 2 Health Sciences Postgraduate Program, Federal University of Rio Grande do Norte (UFRN), Natal 59012-570, RN, Brazil; 3 Specialization Program in Oncotic Gynecological Cytology, Januário Cicco Maternity School, Natal 59012-300, RN, Brazil; 4 Undergraduate Course in Medicine, Federal University of Rio Grande do Norte (UFRN), Natal 59012-570, RN, Brazil; 5 Undergraduate Course in Biomedicine, Federal University of Rio Grande do Norte (UFRN), Natal 59012-570, RN, Brazil; 6 Department of Obstetrics and Gynecology, Federal University of Rio Grande do Norte (UFRN), Natal 59012-570, RN, Brazil; 7 Department of Clinical and Toxicological Analysis, Federal University of Rio Grande do Norte (UFRN), Natal 59012-570, RN, Brazil

**Keywords:** cervical cancer, biomarkers, miRNAs, exosomes

## Abstract

Different studies show that small non-coding RNAs, such as microRNAs (miRNAs) obtained from exosomes, are considered potential biomarkers in several types of cancer, including cervical cancer (CC). Therefore, the present study seeks to present an overview of the role of circulating exosomal miRNAs with the potential to act as biomarkers for the diagnosis and prognosis of CC and to analyze the presence of these miRNAs according to the stage of CC. For this purpose, a review was developed, with articles consulted from the electronic databases MEDLINE/PubMed, Scopus, and Web of Science published between 2015 and 2021. Seven articles were included after a selection of studies according to the eligibility criteria. In addition to the methods used for sample analysis, detection, and isolation of miRNAs in each article, clinical data were also extracted from the patients studied, such as the stage of cancer. After analyzing the network of the seven miRNAs, they were associated with the immune system, CC progression and staging, and cisplatin resistance. With the belief that studies on miRNAs in cervical cancer would have major clinical implications, in this review, we have attempted to summarize the current situation and potential development prospects.

## 1. Introduction

The incidence and mortality rates of cervical cancer (CC) have been substantially reduced with the implementation of cervical cancer screening programs using cytopathological smear tests. Thus, the cytopathological smear test is accepted as the gold standard technique for cervical cancer screening. However, the accuracy of current cervical cancer screening tests is still low [1,2,3,4,5].

The discovery of microRNAs (miRNAs) has opened a new frontier in cancer research and provides an unprecedented potential for the development of miRNA-based biomarkers that can influence downstream genes and signaling pathways [6].

MicroRNAs (miRNAs) are small non-coding regions in 20–22 nucleotide RNAs, which are currently shown to be promising targets for the diagnosis and prognosis of different types of cancer. Their main physiological functions include feedback mechanisms in the protection of essential biological processes, including cell proliferation, differentiation, and apoptosis [7,8]. 

MiRNAs can be found in their free form in the bloodstream or inserted into exosomes (Exo), which are 30–150 nm membrane vesicles of endocytic origin, secreted by most cell types, especially tumor cells, and are important intercellular communicators that act in the transport of packaged molecules, one of which is miRNA. This exosome-induced miRNA transfer is a strategy for genetic exchange between cells and influences the regulation of physiological and pathological processes of many diseases, including cancer. In addition, exosomes can protect miRNAs against adverse storage and handling conditions, keeping them stable. This condition is associated with the ease of detection in biological fluids of this complex, increasing the scientific interest in miRNAs and exosomal miRNAs as potential carcinogenic biomarkers [9,10,11].

Recent advances have proven that not only tumor suppressors and oncogenes but also non-coding RNAs, including micro RNAs (miRNAs), have a significant impact on the development and progression of cervical cancers. Previous studies have identified many cancer-specific miRNAs in the early detection of CC. However, few studies on cervical cancer and exosomal miRNAs have been discovered [11,12]. 

Studies show that miRNA alters the level of expression in cervical cells integrated with HPV, and altered miRNA expression profiles have been reported in CC. Studies investigating cervical cancer cervical wash samples point to an increase in numerous miRNAs such as miRNA-483-5p, miRNA-1246, miRNA-1275, miRNA-21, miRNA-146a and miRNA-222-3p f while some miRNAs such as let-7d-5p, miRNA-92a-3p, miRNA-20a-5p, miRNA-378a-3p, miRNA-423-3p, miRNA-7-5p, miRNA-378c, miRNA-99-5p, miRNA-100 5p and miRNA-320a were down-regulated. [11]

In addition to the functions of exosomes already described, when it comes to cancer, they may also be involved in the process of metastasis, angiogenesis, tumor immunity, and resistance to treatment. At the same time, evidence has recognized miRNAs as diagnostic biomarkers for CC and precancerous lesions [12,13,14]. However, the role of exosomal CC-derived miRNAs as a diagnostic biomarker and their contribution to CC progression has not been fully studied. In this review, we will discuss the role of exosomes and miRNAs in CC and their relationship with the staging of the disease.

## 2. Results

**Figure 1:**
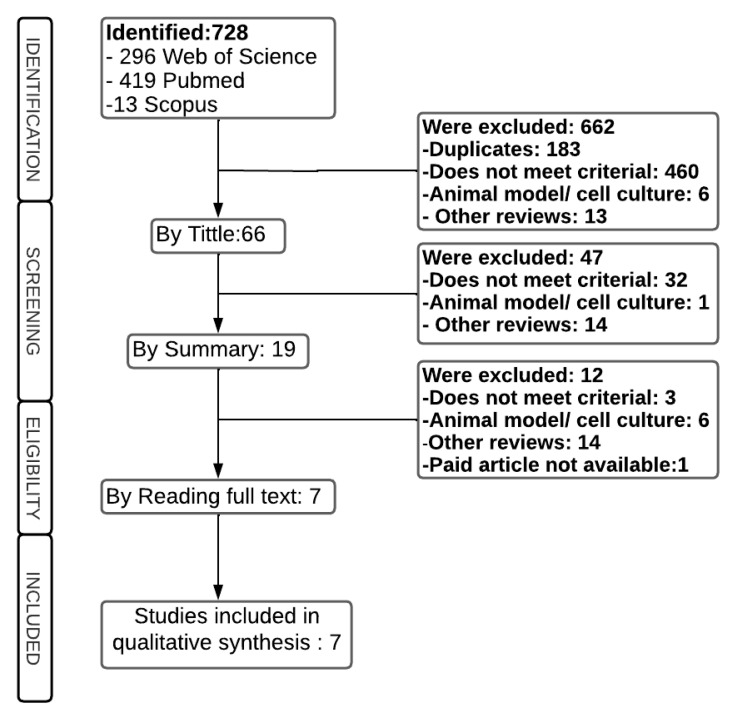
Flowchart of the study selection process.

A total of 728 articles were identified in the databases, according to the inclusion and exclusion criteria described in Section 4.2, of which 662 were excluded at the title reading stage: 183 duplicates, 460 did not meet the criteria, largely because they were about other cancers; 6 involved an animal model or cell culture, and 13 others were reviews. Of the remaining 66 articles, 47 were excluded after screening the abstract of the articles because they included studies that exclusively described cell culture, animal models, articles on other topics, and other reviews. Therefore, 19 articles remained for full reading, and 12 were excluded, leaving 7 articles for the continuation of this review (Figure 1).

### 2.1. Characteristics of the Included Studies

#### 2.1.1. Overall Characteristics

Table 1 presents summarized characteristics of the seven included studies. Data show that about 85% of studies were published in the last 3 years (2019, 2020, and 2021), with all of them published in Asian countries, with China being the most prevalent (71% of published articles). The main type of study was a cross-sectional study, with only one cohort study being observed. The main purpose of the studies was the diagnosis of CC through the use of miRNAs, and no similarities were found between the miRNAs studied in the articles.

#### 2.1.2. Clinical Features

The studies ranged from 184 patients to 28 patients. The characteristics of CC staging were extracted, and different stages of the disease were described in the articles. The most advanced stages were the most prevalent described. The general characteristics of the studies can be seen in Table 1.

#### 2.1.3. Sample Protocols

Most studies used plasma in their analyses, and the exosome and miRNA isolation protocols were different. The use of quantitative reverse transcription PCR (qRT-PCR) was the most commonly used for miRNA detection, and the results are described in Table 2. 

### 2.2. Exosomal miRNAs for the Diagnosis of CC

Clinical research carried out in 2016 by Nagamitsu et al. [15] in Japan, involving MiRNA and CC, extracted the RNA of healthy individuals from the serum (31 patients); individuals with cervical intraepithelial neoplasia (CIN) (55 patients); and patients with cervical cancer (45 patients). The respective study was carried out in three phases. The first and most comprehensive using microarray identified 6 of the 1223 miRNAs found in serum samples from patients with cervical cancer. Normal controls exhibited a >3.0-fold change in the expression level in individuals with cervical cancer. In the second phase (selection phase), the number of miRNAs was decreased from six to four miRNA species (miRNA- 483-5p, miRNA-1275, and miRNA-1290), and a more significant expression was obtained in patients with cervical cancer when compared to healthy controls. In the last phase, the validation was carried out of the four miRNAs mentioned. These last two steps (selection and validation) occurred through polymerase quantitative reverse transcription chain reaction (RT-qPCR). The study decided to follow the other steps with an investigation of miRNA-1290, as it showed an especially greater difference in its expression. It demonstrated that the level of serum miRNA-1290 expression increased along with the stage of cervical cancer. Thus it was reported to be a potential candidate as a biomarker in the diagnosis of uterine cancer.

In 2019, in China, G. Ma et al. [16] obtained plasma samples from 97 CC patients and 87 controls in a multiphase study using qRT-PCR to identify the diagnostic potential of miRNA species. This study highlighted positive regulation in four plasma miRNAs (miRNA-146a-5p, miRNA-151a-3p, miRNA-2110, and miRNA-21-5p). This study also evaluated tumor tissue in smaller amounts justifying that most circulating miRNAs originate from tumor tissues, with the result that levels of miRNA-146a-5p and miRNA-21-5p were all upregulated in CC tissue specimens, while levels of miRNA-146a-5p, miRNA-151a-3p and miRNA-2110 were upregulated in plasma exosomes.

Also in 2019, in the same country, Zheng et al. [17] performed exosomal miRNA sequencing in 121 plasma samples, including 23 volunteers, 5 patients with CIN I, 59 with CIN II-III, 21 with cervical squamous cell carcinoma (SCC) and 13 with adenocarcinoma (ACC). In this step, a total of 312 miRNAs with mean values of log2(RPM + 1) > 1 were detected. The CIN I- samples were used as reference data for comparison with the other sample groups (CIN II-III, CC, SCC, and ACC). This comparison led to the identification of 37 differentially expressed miRNAs (DEmiRs). In another step, the study selected a group of miRNAs from these 37 DEmiRs using the random forest algorithm. This led to the identification of the best panel with eight miRNAs (let-7a-3p, let-7d-3p, miRNA-30d-5p, miRNA- 144-5p, miRNA-182-5p, miRNA-183-5p, miRNA-215-5p and miRNA-4443), which are the strongest predictors in clinical diagnosis. The study ranks itself as one of the largest in the area of miRNA analysis versus cervical cancer, having carried out several steps, which concluded in the end that miRNA-30d-5p and let-7d-3p are valuable diagnostic biomarkers for the non-invasive screening of cervical cancer and its precursors.

In the following year, Lv et al., 2020 [18], conducted a study recruiting 72 individuals, including 44 patients with cervical cancer and 28 healthy controls. The study performed the sequencing phase using 12 samples, 6 patients with CCl and 6 healthy controls. Then, the remaining 60 subjects, which included 22 healthy subjects and 38 CHD patients, were investigated using quantitative PCR (qPCR) to confirm DEmiRs. Further steps were performed, such as ZetaView nanoparticle tracking analysis (NTA), transmission electron microscopy (TEM), and later statistical analyses, which resulted in a total of 1725 unique mature miRNAs being identified from exosome miRNA sequencing data. The results showed 39 miRNAs were differentially expressed between cervical cancer patients and healthy controls, among which 8 miRNAs were down-regulated and 31 miRNAs were upregulated (*p* < 0.001; fold-change > 2.0). The species miRNA-125a-5p was selected for the analysis of its diagnostic potential. The results showed through analysis of the ROC curve that the level of exosomal plasma miRNA-125a-5p was a potential marker to differentiate between non-cervical and cervical cancer, with a ROC area under the curve value of 0.7129 (range 95% confidence (CI)). At the cutoff value of 2.537 for miRNA-125a-5p, the diagnostic sensitivities and specificities of cervical cancer were 59.1% and 84.2%, respectively. 

C. Zhou et al.; 2020 [19], brought a new approach to their immunotherapy study, reporting that high levels of miRNA-142-5p positively correlate with indoleamine 2,3- dioxygenase (IDO) expression in tumor-associated lymphatic vessels in advanced cervical squamous cell carcinoma (CSCC). This intercellular enzyme contributes to immune checkpoint development and promotes tumor progression by attenuating effector T-cell responses. The methodology of the study shows that analyses performed on the serum of patients with CSCC resulted in the exosomal serum levels of miRNA-142-5p being higher in patients with CSCC than in healthy controls, as well as a difference in these levels when comparing the advanced stage with those of patients with early-stage CSSC. Thus, exosomal serum miRNA-142-5p may be a marker to distinguish the different stages of CSCC, therefore contributing to the development of personalized diagnostic strategies for patients with different risks of progression.

More recently, Zhu et al., 2021 [20] investigated the relationship of exosomal miRNA- 651 on cisplatin resistance of cervical cancer. They selected 30 patients with CHD and 30 healthy controls, analyzing the circulating expression of miRNA-651 and its potential for diagnosis via RT-qPCR using plasma samples, with reduced expressions of miRNA-651 being reported in patients with CHD when compared with controls (*p* < 0.0001). The data showed that circulating miRNA-651 had a highly sensitive and accurate ability to diagnose cervical cancer (AU = 0.9050, *p* < 0.0001). In the investigation of cisplatin resistance, the assay used cell culture, and reports indicate that downregulated miR-651 may induce cisplatin resistance in cervical cancer.

### 2.3. Exosomal miRNAs for the Prognosis of CC

This review identified that Cho et al., 2021 [21], in Korea, conducted a pilot study to investigate the log 2-fold change (log 2 FC) in the expression of exosomal miRNAs and related messenger RNAs (mRNAs) in samples from patients with breast and cervix cancer to identify better prognostic markers than currently available. This study explored important points, such as the stage of CC, data from patients before and after the start of chemotherapy, and miRNA-mRNA interactions in early progression. Plasma exosomal RNA sequencing and profiling steps were performed on 29 samples from previously diagnosed CHD patients, having analyzed 586 miRNAs and 15,324 mRNAs in the respective samples.

**Figure 2:**
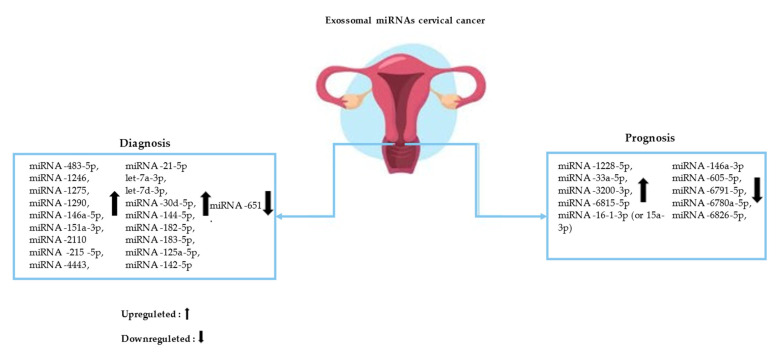
Circulating exosomal microRNAs (miRNAs) and their action in the diagnosis and prognosis of cervical cancer (CC).

This research demonstrated reduced levels of miRNA-1228-5p, miRNA-33a-5p, miRNA-3200-3p, and miRNA-6815-5p, and increased levels of miRNA-146a-3p in patients with early progression and also revealed unresolved inflammation. Whereas increased levels of miRNA- 605-5p, miRNA-6791-5p, miRNA-6780a-5p, and miRNA-6826-5p and decreased levels of miRNA-16-1-3p (or 15a-3p) were associated with the degree of metastasis. They led to the systemic activation of myeloid, endothelial, and epithelial cells, as well as neurons, phagocytes, and platelets. The Log 2 FCs in the expression of miRNAs and mRNAs from plasma exosomes after treatment are associated with early progression and metastasis, reflecting unresolved inflammation and systemic microenvironmental factors, respectively [21] (Figure 2).

### 2.4. Exosomal miRNAs Related to CC Staging

Taking into account that the survival rates of patients with carcinoma are different between metastatic and non-metastatic, there is a need to classify the stages of cancer, which consists of analyzing its degree of dispersion between organs. For this, there are different classification systems, one of which is the International Federation of Gynaecologists and Obstetricians (FIGO), which has a staging system for gynecological cancers, including CC [22,23].

Nagamitsu et al. [15] reported indices of serum miRNA-1290 expressions according to the different stages of CC, demonstrating the increase through RT-PCR analysis and the Kruskal–Wallis test (*p* < 0.01) of these levels. In each set of patients studied, the healthy group, 0.78; CIN group 1–2, 1.81; CIN3 group, 2.74; stage I cervical cancer group, 4.00; stage II group, 5.66; and stage III–IV group, 5.59.

G. Ma et al. [16] studied the clinical characteristics of patients, including the histological grade and FIGO stage of the disease, reporting higher miRNA-151a-3p expression values in histological grade I patients when compared with a higher grade of disease (grade II or III), as well as an increase in miRNA-151a-3p in patients with stage I disease in the FIGO classification compared to those with stage II disease. The expression of miRNA-21-5p was also analyzed, showing an increase in its plasma levels in patients with grade II or III disease compared to those with grade I disease. It was further reported that miRNA-146a-5p expression was higher in adenocarcinoma than in squamous cell carcinoma. While Lv et al. [18], when studying miRNA-125a-5p, found no difference in the expression of this miRNA between cervical squamous cell carcinoma and adenocarcinoma (AC). The other stages of CC were analyzed using Mean ± SD, being described in this study as 29 patients in stage IA–IIA (0.95 ± 0.59) and 9 patients in stage IIB–IVB (1.60 ± 0.94).

C. Zhou et al. [19] examined the levels of miRNA-142-5p in patients with stages I and II (initial stage—FIGO) and patients with stages III and IV (advanced stage-FIGO) with CSCC and healthy controls. They identified that exosomal serum values of miRNA-142-5p in early stages were lower than in patients with advanced stage, and levels of miRNA-142-5p were higher in patients with CSCC than in healthy controls.

Zheng et al. [17] reported a broad description of miRNAs according to each cervical cancer group and precursor lesions. About 27 types of miRNAs were cataloged in this study of two groups (NIC II-III and CC). In total, 12 types of miRNAs were present in patients with CSCC, and 8 types were present in patients with AC, significantly 4 miRNAs (let-7a-3p; let-7d-3p; miRNA-144-5p; miRNA-30d-5p) were present in all groups (NIC II-III, CC, CSCC, and AC). The study reported that the presence of these types of miRNAs: let-7a-3p, let- 7d-3p, miRNA-30d-5p, miRNA-144-5p, miRNA-182- 5p, miRNA-183-5p, miRNA-215-5p, and miRNA-4443, are strong indicators of CIN I- (low lesion grade) and CIN II+ (lesion high-grade and ASC-H), regardless of HPV types.

Cho et al. [21] described the clinical data of 18 studied patients, classified according to the FIGO scale, in which those with stage IB-IIIC1 had their lesions located in the pelvis, those with stage IVB they had spread to the lungs and supraclavicular lymph nodes(LNs) and axillary. Those with stage IIIC2-IVA spread to the popliteal lymph nodes(PALNs). The stages were associated with the presence of miRNA-605-5p, miRNA-6791-5p, miRNA-6780a-5p, miRNA-6826-5p, and miRNA-16- 1-3p (or 15a-3p). The advanced stages for distant metastasis from the pelvis were negatively linked with miRNA-16-1-3p (or 15a-3p) but positively correlated with miRNA-605-5p, miRNA-6791-5p, miRNA-6780a-5p, and miRNA-6826-5p.

### 2.5. Limitation

The lack of robust studies on the scope of miRNAs is considered a limiting factor of this study. Most of them were in Asia, so research on other populations needs to be conducted. There is a lack of homogeneity in the study designs and in the molecules found, and it is identified that the methods of detection of circulating miRNAs need to be improved. Larger samples are suggested, and more research will be needed to identify reliable circulating exosomal miRNAs for use as biomarkers in the early diagnosis and prognosis of patients with CC as well as to discover their relationship to the stages of this disease. Therefore, comprehensive studies that provide important additional information are lacking.

## 3. Discussion

Cervical cancer is a pathology with a high number of deaths among women, and ways that favor its identification, progression, and treatment are essential to improve survival among women. Therefore, studies of miRNA offer a promising route to improvements [24,25]. The use of miRNAs is key to a gradual increase in research over the forthcoming years. Existing research is still ongoing on the characterization of miRNA profiles within different tumor subtypes. There are questionable delays that delay the exemption of techniques with the use of miRNAs from bench to bedside [9].

As observed in our results, there is no consensus on potential miRNAs that can be used in clinical practice. That is, the different studies do not show common miRNAs among them. Several miRNAs were found to be out of balance by different groups using different methodologies, such as Zheng et al. [17], who observed in their study the presence of 312 different types of miRNA in samples from patients at different stages of CC. This range of miRNAs may be one of the justifications for the high heterogeneity of the findings of this review.

There was also a predominance of the use of plasma samples and qRT-PCR as a means of miRNA detection. This is the main method used in this phase due to its sensitivity and specificity, which corroborates other studies [9,10,11,12,13,14,15,16,17,18,19,20,21,22,23,24].

In one study, serum miRNA-1290 increased along with the stage of cervical cancer. The discussion reported that this miRNA was present in chromosome 1:19,223,565–19,223,642. Further, it noted that B-cell lymphoma 2 (BCL2) and microtubule-associated tau protein (MAPT) are potential targets of miRNA-1290 according to in silico analysis [15,26]. Moreover, another study reported that miRNA-146a-5p and miRNA-21-5p were all upregulated in CC tissue samples, while levels of miRNA-146a-5p, miRNA-151a-3p and miRNA-2110 were upregulated in plasma exosomes. Serum miRNA-146a-5p was previously reported to be involved in the pathogenesis of CC. At the same time, miRNA-146a has been shown to promote CC cell viability by targeting IRAK1 (Interleukin 1 Receptor Associated Kinase 1) and TRAF6 (TNF Receptor Associated Factor 6) [16,17,18,19,20,21,22,23,24,25,26,27]. Other studies have shown that miRNA-30d-5p, let-7d-3p, miRNA-125a-5p, and miRNA-142-5p are potential candidates as a biomarker in the diagnosis of CC [17].

miRNA-146a-5p belongs to the miRNA-146a family [28], which in turn was reported in two other reviews dealing with colorectal cancer (CRC) and CC, respectively, both of which were upregulated. The first study describes large amounts of exosomal miRNA-146a in circulation correlated with low levels of CD8 T cells infiltrating the tumors, and high levels of CD66 neutrophils, suggesting that this miRNA is the main exosome secreted by colorectal cancer stem cells. Meanwhile, the second review described the presence of this miRNA associated with the presence of types 16, 18, 31, and 68/HPV. Another miRNA found in both reviews was miRNA-21, of which miRNA-21-5p belongs to its sequence. miRNA-21 was shown to be associated with liver metastases and tumor staging in patients with RCC in plasma samples and is upregulated in these patients. In another six articles found in the review involving CC, miRNA-21 was also upregulated, being more associated with the presence of HPV 16 [9,10,11,12,13,14,15,16,17,18,19,20,21,22,23,24]. Two other miRNAs were found in common with our review and also a study dealing with CCR, which reported the presence of upregulated miRNA-1290 for diagnostic purposes and upregulated miR-30d-5p for surveillance and follow-up purposes [9].

Apparently, there is still a relationship between the expression levels of certain miRNAs when compared with the stages/grade of the disease. One of the main studies found in this review identified four types of miRNAs present in all stages of CC, these being let-7a-3p; let-7d-3p; miRNA-144-5p, and miRNA-30d-5p [17]. In advanced stages of the disease, one of the studies showed an association between the presence of miRNA-605-5p, miRNA-6791-5p, miRNA-6780a-5p, miRNA-6826-5p, and miRNA-16-1-3p (or 15a-3p), advanced stages of distant pelvic metastases were negatively linked with miRNA-16-1-3p (or 15a-3p) while being positively correlated with miRNA-605-5p, miRNA-6791-5p, miRNA-6780 a-5p, and miRNA-6826-5p. [25]. One of the studies reported the action of exosomal levels of miRNA-651 related to resistance to cisplatin, one of the main drug treatments currently used, in addition to being downregulated in CC, considered in this study as a potential diagnostic marker as well as a potential therapeutic agent [20].

Larger studies are essential to guide therapeutic, diagnostic, and prognosis patterns of CC. The limitations of this study need to be minimized to obtain better results. However, the results already found are promising and point to an encouraging perspective on the use of miRNAs in clinical practice.

## 4. Materials and Methods

### 4.1. Bibliographic Research Strategy

We performed a review of original articles from the current literature (2015 to 2021) using the following bibliographic search databases MEDLINE/PubMed, Scopus, and Web of Science, using a combination of the following Medical Subject Headings (MESH) terms and keywords: “cervical cancer”, “Uterine Cervical Neoplasms”, “exosome”, “plasma”, “serum”, “microRNA” “miRNA” and “miR”.

### 4.2. Selection of Studies: Inclusion and Exclusion Criteria

Inclusion criteria were studies published during the established period, selected according to title and abstract, that evaluated the potential of exosomal plasma/serum miRNAs for use as biomarkers for the diagnosis, prognosis, and pathogenicity of CC. Exclusion criteria were studies with animal models, studies based exclusively on cell culture, brief communications, other analyzes on the subject, paid articles not available on the web, and bioinformatics analyses.

### 4.3. Data Review and Extraction Section

To review the research results, four different authors were used. All studies were listed in the EndNote software (EndNote X7, Thomson Reuters), then distributed among the authors for evaluation. Each study was analyzed by two independent authors, and the third expert author strategy was used for conflicts. In the review, we investigated the study design and its eligibility based on the inclusion criteria. All original research studies in each study setting refer to the diagnosis and/or prognosis of patients with CHD through miRNAs, regardless of the patient population or age group considered for the screening and review section. The name of the first author, country of the study performed, year of publication, sample size, type of miRNA, as well as the method of detection and isolation, study design, and CC staging, were extracted from all primary studies included.

### 4.4. Bias Assessment

The risk of bias was analyzed using the JBI Levels of Evidence [29], with the observational studies/descriptive studies classified according to the tool as being level 4.6, while the only cohort study found in this review was classified as level 3.b.

## 5. Conclusions

Considering the severity of cervical cancer and the need to develop new means of diagnosis, prognosis, and monitoring of the disease, the study of miRNAs for these purposes proved to be promising in this review. However, larger studies are needed to guide therapeutic, diagnostic, and prognosis patterns of CC.

## Figures and Tables

**Table 1:** Clinical features, types of studies, and types of miRNAs.

Authors and Year	Country	Design of Study	Number of Patients	Stage of Cervical Cancer	Type of miRNA	Prognosis or Diagnosis	Results
Nagamitsu et al., 2016	Japan	Cross sectional	45 had cervical cancer, 55 had NIC, and 31 were healthy.	Of the 45 patients with cervical cancer, 7 were stage Ia, 16 were stage Ib, 10 were stage IIb, 3 were stage III and 2 were stage IV.	miRNA-485-5p, miRNA-1246, miRNA-1275, miRNA-1290	Diagnosis	The circulating serum miRNA-485-5p, miRNA-1246, and miRNA-1275, as well as miRNA-1290, were significantly higher in subjects with cervical cancer compared to healthy controls, the expression of circulating miRNA-1290 was significantly higher in the blood of patients with cervical cancer compared to controls. It may thus serve as a useful biomarker in the diagnosis of cervical cancer. However, larger studies are needed to fully elucidate the role of circulating exosomal miRNAs in cervical cancer.
G. Ma et al., 2019	China	Cross sectional	97 CC patients and 87 NCs	FIGO I—74/ FIGO II—23	miRNA-146a-5p, miRNA- 151a-3p, miRNA-2110, miRNA-21-5p	Diagnosis	Four plasma miRNAs (miRNA-146a-5p, miRNA-151a-3p, miRNA-2110 and miRNA-21-5p) that showed upregulation were identified and validated in patients with CC. A panel of the four miRNAs was constructed as potential diagnostic markers for CC. The levels of miRNA-146a-5p and miRNA-21-5p were all upregulated in CC tissue samples, while the levels of miRNA-146a-5p, miRNA-151a-3p and miRNA-2110 were upregulated in plasma exosomes in cervical cancer subjects compared with healthy controls
Lv et al., 2020	China	Cross sectional	72	Not described	miRNA-125a-5p	Diagnosis	The results showed through analysis of the ROC curve that the level of exosomal plasma miRNA-125a-5p was a potential marker to differentiate between non-cervical and cervical cancer.
Cho et al., 2021	Korea	Cohort	28	CC IB-IVB	miRNA-1228-5p, miRNA- 146a-3p, miRNA-33a-5p, miRNA-3200-3p, miRNA-501-3p, miRNA-6815-5p	Prognosis	The log2FCs of miRNAs and mRNAs from plasma exosomes were found to be associated with unresolved inflammation and microenvironmental factors that trigger metastasis.
C. Zhou et al.; 2020	China	Cross sectional	116 human and stages III and IV, *n* = 23 were analyzed.	(FIGO 2018, stages I and II, *n* = 27) and advanced-stage (FIGO 2018, stages III and IV, *n* = 17)	miRNA-142-5p	Diagnosis	CSCC malignant progression altered the level of miRNA- 142-5p in serum exosomes, indicating that serum exosomal miRNA-142-5p may discriminate between indolent and aggressive CSCC and contribute to the development of personalized diagnostic strategies for patients with different progression risks.
Zhu et al., 2021	China	Cross sectional	30	not described	miRNA-651	Diagnosis	Collectively, this study showed that cancer-derived exosomal miRNA-651 may restrain cisplatin resistance and progression and directly target ATG3 in cervical cancer. Hence, exosomal miR-651 could be a therapeutic agent against cervical cancer.
Zheng et al., 2019	China	Cross sectional	121	NIC I/NIC II +/ ACC and SCC	A total of 312 miRNAs with mean log2(RPM + 1) values >1 were detected from miRNA sequencing of exosomes derived from 121 plasma samples	Diagnosis	The present study represents one of the largest plasma miRNA studies for cancer biomarker discovery. The identified exosomal miRNA-30d-5p and let-7d-3p are valuable diagnostic biomarkers for non-invasive screening of cervical cancer and its precursors. Blood extraction is more convenient and carries less risk of vaginal/uterine cervix infection than TCT or Pap smear tests.

Subtitle: NCs: normal controls, CC: cervical cancer, NIC: cervical intraepithelial neoplasia, ACC: adenocarcinoma, SCC and CSCC: cervical squamous cell carcinoma, FIGO: International Federation of Gynecologists and Obstetricians.

**Table 2:** Characteristics of sample protocols, isolation methods, detection and validation of exosomal miRNAs.

Authors and Year	Sample Type	Sample Processing Conditions	Method of Exosome Isolation	Method of RNA/miRNA Isolation	Method of miRNA Detection
Nagamitsu et al., 2016	Serum	The samples were separated into blood cells and serum by centrifugation and stored at −5 °C.	Not descriptive	Total RNA in the serum was isolated using ISOGEN-LS, according to the manufacturer’s instructions (NIPPON GENE CO., LTD., Toyama, Japan).	Microarray and RT-qPCR
G. Ma et al., 2019	Plasma	Plasma samples were clarified, spinning at 350 g for 10 min at 4 °C, followed by 20,000× *g* for 10 min at 4 °C and then stored at −80 °C until use.	Exosomes were extracted from plasma following the manufacturer’s instructions for the Exo-Quick exosome precipitation solution (System Biosciences, Mountain View, CA, USA).	RNA was isolated from 200 µL of plasma using the mirVana Paris kit (Ambion, Austin, TX, USA) and Trizol (TaKaRa, Dalian, China) according to recommended conditions.	qRT-PCR
Lv et al., 2020	Plasma	Samples were centrifuged at 800× *g* for 15 min at 4 °C	exoEasy Maxi kit (cat. no. 76064; Qiagen, Inc.) according to the manufacturer’s instructions	Kit miRNeasy Serum/Plasma (cat. n.° 77064; Qiagen, Inc., Hilden, Germany)	qRT-PCR (LineGene K Plus; Hangzhou Bioer Co., Ltd., Hangzhou, China)
Cho et al., 2021	Plasma	Not descriptive	conducted by Macrogen (Seoul, Republic of Korea)	Not descriptive	Next generation sequencing data
C. Zhou et al.; 2020	Serum	All blood samples were centrifuged at 2500× *g* for 10 min to extract serum. All samples were stored at –80 °C until further study.	Ultracentrifugation	miRNeasy Micro Kit (Qiagen) was used according to the manufacturer’s instructions. Specific primer sets for miR-142-5p and U6 were obtained from RiboBio Inc. The expression of miRNAs and mRNAs was normalized to U6 and GAPDH, respectively.	RT-qPCR
Zhu et al., 2021	Plasma	5 mL whole blood samples were harvested from each subject by using EDTA anticoagulation tube. Following centrifugation and separation, samples were collected in an EP tube and stored at −80 °C for later use.	Exosome-Free Serum Preparation. FBS was centrifuged at 100,000× *g* for 70 min, and the precipitate was removed to obtain exosome-free FBS. HeLa/S cells were cultured with RPMI 1640 medium (Hyclone, Logan, UT, USA) with exosome-free 10% FBS.	TRIzol (Invitrogen, USA)	qRT-PCR
Zheng et al., 2019	Plasma	The plasma samples were centrifuged at 16,000× *g* for 10 min at 4 °C before storage at −80 °C until use.	Exosomes were isolated using an ExoQuick exosome precipitation solution. (SBI Cat#:100356EXOQ20A-1, Mountain View, CA, USA) mixed with RNase A (Sigma Cat#: R6513-10MG, St. Louis, MO, USA)	miRNeasy Micro Kit (QIAGEN Cat#:217084, Valencia, CA, USA)	qRT-PCR and digital PCR (ddPCR)

## Data Availability

Not applicable.

## References

[1] Cohen A.P., Jhingran A., Oaknin A., Denny L. (2019). Cervical cancer. Lancet.

[2] William W., Ware A., Basaza-Ejiri A.H., Obungoloch J. (2019). A pap-smear analysis tool (PAT) for detection of cervical cancer from pap-smear images. Biomed. Eng. Online.

[3] Mahmoodi P., Fani M., Rezayi M., Avan A., Pasdar Z., Karimi E., Amiri I.S., Ghayour-Mobarhan M. (2019). Early detection of cervical cancer based on high-risk HPV DNA-based genosensors: A systematic review. Biofactors.

[4] Adem K., Kiliçarslan S., Cömert O. (2019). Classification and diagnosis of cervical cancer with stacked autoencoder and softmax classification. Expert Syst. Appl..

[5] Philp L., Jembere N., Wang L., Gao J., Maguire B., Kupets R. (2018). Pap tests in the diagnosis of cervical cancer: Help or hinder?. Gynecol. Oncol..

[6] Weng W., Feng J., Qin H., Ma Y., Goel A. (2015). An update on miRNAs as biological and clinical determinants in colorectal cancer:A bench-to-bedside approach. Future Oncol..

[7] Reddy K.B. (2015). MicroRNA (miRNA) in cancer. Cancer Cell Int..

[8] Khan A.Q., Ahmed E.I., Elareer N.R., Junejo K., Steinhoff M., Uddin S. (2019). Role of miRNA-regulated cancer stem cells in the pathogenesis of human malignancies. Cells.

[9] Santos K.A., Santos I.C.C., Silva C.S., Ribeiro H.G., Domingos I.F., Silbiger V.N. (2021). Circulating exosomal miRNAs as biomarkers for the diagnosis and prognosis of colorectal cancer. Int. J. Mol. Sci..

[10] Madhavan B., Yue S., Galli U., Rana S., Gross W., Müller M., Giese N.A., Kalthoff H., Becker T., Büchler M.W. (2015). Combined evaluation of a panel of protein and miRNA serum-exosome biomarkers for pancreatic cancer diagnosis increases sensitivity and specificity. Int. J. Cancer.

[11] Nahand J.S., Vandchali N.R., Darabi H., Doroudian M., Banafshe H.R., Moghoofei M., Babaei F., Salmaninejad A., Mirzaei H. (2020). Exosomal microRNAs: Novel players in cervical cancer. Epigenomics.

[12] Liu S.S., Chan K.K.L., Chu D.K.H., Wei T.N., Lau L.S.K., Ngu S.F., Chu M.M.Y., Tse K.Y., Ip P.P.C., Ng E.K.O. (2018). Oncogenic micro RNA signature for early diagnosis of cervical intraepithelial neoplasia and cancer. Mol. Oncol..

[13] Bhat A., Sharma A., Bharti A.C. (2018). Upstream Hedgehog signaling components are exported in exosomes of cervical cancer cell lines. Nanomedicine.

[14] Zhang L., Li H., Yuan M., Li M., Zhang S. (2019). Cervical cancer cells-secreted exosomal microRNA-221-3p promotes invasion, migration and angiogenesis of microvascular endothelial cells in cervical cancer by down-regulating MAPK10 expression. Cancer Manag. Res..

[15] Nagamitsu Y., Nishi H., Sasaki T., Takaesu Y., Terauchi F., Isaka K. (2016). Profiling analysis of circulating microRNA expression in cervical cancer. Mol. Clin. Oncol..

[16] Ma G., Song G., Zou X., Shan X., Liu Q., Xia T., Zhou X., Zhu W. (2019). Circulating plasma microRNA signature for the diagnosis of cervical cancer. Cancer Biomark..

[17] Zheng M., Hou L., Ma Y., Zhou L., Wang F., Cheng B., Wang W., Lu B., Liu P., Lu W. (2019). Exosomal let-7d-3p and miR-30d-5p as diagnostic biomarkers for non-invasive screening of cervical cancer and its precursors. Mol. Cancer.

[18] Lv A., Tu Z., Huang Y., Lu W., Xie B. (2021). Circulating exosomal miR-125a-5p as a novel biomarker for cervical cancer. Oncol. Lett..

[19] Zhou C., Zhang Y., Yan R., Huang L., Mellor A.L., Yang Y., Chen X., Wei W., Wu X., Yu L. (2021). Exosome-derived miR-142-5p remodels lymphatic vessels and induces IDO to promote immune privilege in the tumour microenvironment. Cell Death Differ..

[20] Zhu X., Long L., Xiao H., He X. (2021). Cancer-Derived Exosomal miR-651 as a Diagnostic Marker Restrains Cisplatin Resistance and Directly Targets ATG3 for Cervical Cancer. Dis. Markers..

[21] Cho O., KIM D.W., Cheong J.Y. (2021). Plasma Exosomal miRNA Levels after Radiotherapy Are Associated with Early Progression and Metastasis of Cervical Cancer: A Pilot Study. J. Clin. Med..

[22] World Health Organization Classification TNM/FIGO. https://screening.iarc.fr/atlasclassiftnm.php?lang=4>.

[23] American Cancer Society Cancer Staging. https://www.cancer.org/treatment/understanding-your-diagnosis/staging.html.

[24] Nahand J.S., Taghizadeh-boroujeni S., Karimzadeh M., Borran S., Pourhanifeh M.H., Moghoofei M., Bokharaei-Salim F., Karampoor S., Jafari A., Asemi Z. (2019). microRNAs: New prognostic, diagnostic, and therapeutic biomarkers in cervical cancer. J. Cell. Physiol..

[25] Shen S., Zhang S., Liu P., Wang J., Du H. (2020). Potential role of microRNAs in the treatment and diagnosis of cervical cancer. Cancer Genet..

[26] Endo Y., Toyama T., Takahashi S., Yoshimoto N., Iwasa M., Asano T. (2013). miR-1290 and its potential targets are associated with characteristics of estrogen receptor α-positive breast cancer. Endocr.-Relat. Cancer.

[27] Hu Q., Song J., Ding B., Cui Y., Liang J., Han S. (2018). miR 146a promotes cervical cancer cell viability via targeting IRAK1 and TRAF6. Oncol. Rep..

[28] Paterson M.R., Kriegel A.J. (2017). MiR-146a/b: A family with shared seeds and different roots. Physiol. Genomics.

[29] Joanna Briggs Institute and University of Adelaide (2014-Updated May 09, 2022) New JBI Levels of Evidence. https://ospguides.ovid.com/OSPguides/jbidb.htm.

